# Research Progress of RNA Methylation Modification in Colorectal Cancer

**DOI:** 10.3389/fphar.2022.903699

**Published:** 2022-05-03

**Authors:** Weizheng Liang, Hongyang Yi, Chenyu Mao, Qingxue Meng, Xueliang Wu, Shanliang Li, Jun Xue

**Affiliations:** ^1^ Central Laboratory, The First Affiliated Hospital of Hebei North University, Zhangjiakou, China; ^2^ The Third People’s Hospital of Shenzhen, Shenzhen, China; ^3^ School of Engineering and Applied Science, University of Pennsylvania, Philadelphia, PA, United States; ^4^ Department of General Surgery, The First Affiliated Hospital of Hebei North University, Zhangjiakou, China; ^5^ Department of Pharmacology, Guangxi University of Chinese Medicine, Nanning, China

**Keywords:** colorectal cancer, RNA modification, RNA methylation, N6-methyladenosine, 5-methylcytosine, N1-methyladenosine, 7-methylguanine, circular RNA

## Abstract

Accumulating evidence indicates that RNA methylation, as the most common modification of mRNA, is of great significance in tumor progression and metastasis. Colorectal cancer is a common malignant tumor of the digestive system that seriously affects the health of middle-aged and elderly people. Although there have been many studies on the biological mechanism of the occurrence and development of colorectal cancer, there are still major deficiencies in the diagnosis and prognosis of colorectal cancer. With the deep study of RNA methylation, it was found that RNA modification is highly related to colorectal cancer tumorigenesis, development and prognosis. Here, we will highlight various RNA chemical modifications including N6-methyladenosine, 5-methylcytosine, N1-methyladenosine, 7-methylguanine, pseudouridine and their modification enzymes followed by summarizing their functions in colorectal cancer.

## Introduction

With the fourth modality rate among all diseases, colorectal cancer (CRC) is one of the most commonly seen tumors of the gastrointestinal tract, and its incidence is the highest in developed countries (Brody, 2015; Bray et al., 2018; Siegel et al., 2020). At present, colorectal cancer is gradually tending to be younger (Akimoto et al., 2021). The pathogenesis of colorectal cancer is complex and diverse, which may be led by an unhealthy diet, obesity, lack of exercise, and microbial infection. Long-term exposure to these risk factors affects the intestinal microbiota and host immunity, resulting in genetic and epigenetic alterations in colorectal epithelial cells, ultimately predisposing them to colorectal cancer (Brody, 2015; Akimoto et al., 2021). The progression of colorectal tumors from adenoma to colorectal cancer is a multi-step pathological process of tumorigenesis that takes about 10 years. Abnormal mutations, accumulation of proto-oncogenes and tumor suppressor genes play a huge part in this process which includes heritable changes in the genome with changes in the basic nucleotides of DNA and stable inheritance of unchanged nucleotide sequences that cause changes in gene expression and function, that is, epigenetics (Sjöblom et al., 2006; Markowitz and Bertagnolli, 2009).

Epigenetic modifications are dynamically reversible and heritable, including DNA methylation, histone modifications, chromatin remodeling, microRNAs (miRNAs) and noncoding RNAs (ncRNAs) which are of great importance in the progression and metastasis of colorectal cancer (Huang et al., 2021a). Recently, major breakthroughs have been made in the research on DNA omics and proteomics in the occurrence and development of tumors, and the epigenetic modification at the RNA level has also attracted the attention of many researchers (Skvortsova et al., 2019; Sivanand and Vander Heiden, 2020; Mobet et al., 2022; Wishart, 2022).

RNA modifications play a key regulatory role in gene expression (Helm and Motorin, 2017; Wu et al., 2021). In recent years, over 170 RNA chemical modifications have been discovered, involving both coding and noncoding RNAs (Boccaletto et al., 2022). In eukaryotic, the most abundant modification found is N6-methyladenosine (m6A), which is considered as eukaryotic characteristic internal modification since its discovery in the 1870s (Gilbert et al., 2016; Davalos et al., 2018). RNA is not only an intermediate or effector molecule in protein synthesis but also has direct functional effects on gene expression through a variety of other noncoding RNAs. Dynamic modification of RNA enables cells to respond rapidly to changes in the external environment, and the ability to adapt to changing microenvironments (such as stimuli and stress) is critical for tumor cell survival. Recent research has demonstrated that RNA modification has become a main emerging regulator in cancer by regulating various RNA metabolic processes (Cui et al., 2017; Barbieri and Kouzarides, 2020; Miano et al., 2021). More and more evidence has shown that the abnormal expression of various m6A regulatory proteins plays a role in promoting or suppressing tumors in human tumors (Nombela et al., 2021). Abnormal changes in RNA modification are often functionally related to cell proliferation, differentiation, stress adaptation, invasion and resistance to chemotherapy. Therefore, targeting abnormal RNA modifications in cancer cells is expected to be an effective way to treat tumors.

In this review, we will summarize the biological characteristics of various RNA modifications, including m6A, m5C, N1-methyladenosine, 7-methylguanine, pseudouridine and their roles in the occurrence and development of colorectal tumors by providing a basis for further search for biomarkers and therapeutic targets.

### m6A Modification and Colorectal Cancer

**FIGURE 1:**
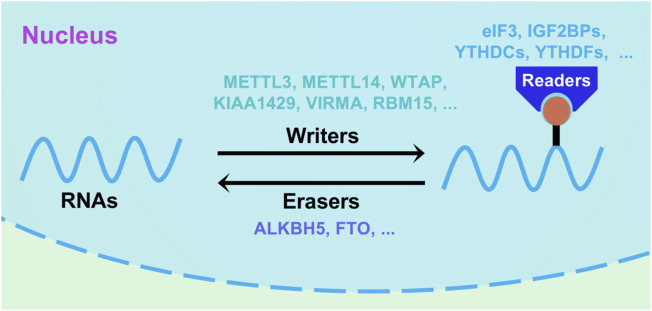
Schematic diagram of RNA m6A methylation mechanism. m6A modification is a dynamic and reversible process, which could be regulated by writers (WTAP, METTL3/14, KIAA1429, VIRMA and RBM15), erasers (FTO and ALKBH5) and readers (eIF3, YTHDCs, YTDFs and IGF2BPs).

m6A methylation modification is a common RNA methylation modification on the 6th N of adenine. It is a dynamically reversible post-transcriptional modification that is most abundant in mRNA and noncoding RNA (Figure 1). The modification of m6A occurs mainly in the RRACH sequence motif (where R = A or G, H = A, C, or U) and is significantly enriched in the mRNA 3′UTR as well as CDS regions (Dominissini et al., 2012; Meyer et al., 2012). m6A is involved in multiple processes of RNA metabolism, including post-transcriptional splicing, translation efficiency and stability maintenance, and is essential in normal physiological processes such as growth and development, learning and memory, and is also involved in regulating the response to body heat shock (Zhou et al., 2015; Shi et al., 2018; Wang et al., 2018). It is also crucial for tumor development and tumor immune drug resistance (Han et al., 2019). m6A modification is regulated by methyltransferases, demethylases, and methyl-recognition proteins as a post-translational RNA modification. Among them, methyltransferase catalyzes the modification of adenylate by m6A in RNA, which is composed of various proteins such as METTL3 (methyltransferase like 3), METTL14 (methyltransferase like 14), WTAP (Wilms tumor 1 associating protein) (Frye et al., 2018; Shi et al., 2019) and KIAA1429 (Liu et al., 2014). The core proteins of demethylase include FTO (fat mass and obesity-associated protein) and ALKBH5 (Alkb homolog 5), which can demethylate bases that undergo a modification of m6A, which is also the cause of dynamic reversibility. Methylation recognition proteins can recognize and bind to m6A-modified bases, and regulate biological processes such as RNA degradation and stabilization, nuclear export, and translation efficiency. Given their functional characteristics, these proteins are called “writer,” “eraser,” and “reader” (Zaccara et al., 2019).

In recent years, m6A modification has gradually become the hot field in studying colorectal tumorigenesis, development and metastasis. m6A modifications have widely altered the expression of certain colorectal tumor-associated genes. Abnormally expressed writer protein, eraser protein, and reader protein, by regulating the level of m6A in different RNAs, affects the function of downstream pathways, such as the classical pathway related to colorectal tumorigenesis and apoptosis, the Wnt pathway and the Hippo pathway, and plays pro- or anti-cancer effects (Chen et al., 2020a; Xu et al., 2020a; Chen et al., 2020b).

It was found that METTL3 expression was increased in tumor tissues of colorectal cancer patients, and the higher its expression, the worse the prognosis of patients (Zhou et al., 2021a; Chen et al., 2021). METTL3 regulates the expression of various oncogenes and tumor suppressor genes at the post-transcriptional level to enhance the progression of colorectal cancer. Overexpression of METTL3 represses SOCS2 and promotes LGR5 promoter activity to maintain cell proliferation in colon cancer cells (Xu et al., 2020b). Knockdown of METTL3 can inhibit the development of colorectal cancer significantly. Research have suggested that METTL3 targets the YPEL5 m6A modification site to inhibit its expression and promote the progression of colorectal cancer (Zhou et al., 2021a). On the other hand, METTL3 stabilizes its expression level by methylating the m6A site of the CCNE1 3′UTR mRNA to increase the expression of the cyclin E1 to promote colorectal cancer cell proliferation (Zhu et al., 2020). Furthermore, a group found that the m6A methyltransferase METTL3 promoted the malignant proliferation of colorectal cancer cells by directly or indirectly upregulating MYC expression (Xiang et al., 2020). In an indirect mechanism, it was found that METTL3 may upregulate MYC expression through the WNT, TGF-β and other signaling pathways. The expression of MYC was also found to be recognized by IGF2BP1 and its stability was enhanced dependent on m6A-IGF2BP1. In addition, METTL3 increases the expression of PTTG3P through an m6A/IGF2BP2-dependent mechanism and regulates the PTTG3P/YAP1 axis to induce colon cancer cell proliferation and promote colon cancer progression (Zheng et al., 2021). METTL3 also regulates the progression of colorectal cancer by affecting the metabolism of tumor cells. Shen et al. found that the m6A methyltransferase METTL3 regulates the expression levels of HK2 and SLC2A1 by regulating m6A expression and stabilizing m6A under the action of the methyl-binding protein IGF2BP, which further promotes the activation of the glycolytic pathway, inducing malignant proliferation of colorectal cancer cells (Shen et al., 2020). Another study has confirmed that METTL3 carries out m6A modification on GLUT1 mRNA to improve its mRNA stability and translation level, and promote glucose metabolism and colorectal cancer occurrence (Chen et al., 2021). Through the regulation of the m6A-GLUT1-mTORC1 axis, METTL3 is actively involved in the proliferation of colorectal cancer, and simultaneous inhibition of mTORC1 and METTL3 has an additive effect on the treatment of colorectal cancer. In addition to the effects on cell proliferation, METTL3 also has relations with the progression and metastasis of colorectal cancer. Peng et al. (2019) demonstrated that the overexpressed m6A methyltransferase METTL3 in colorectal cancer can increase miRNA-1246 expression by upregulating the methylation level of pri-miR-1246, and further lead to tumor metastasis by downregulating the expression of the metastasis-related suppressor gene SPRED2. In addition, it was found that SPRED2 regulates colorectal cancer metastasis through the MAPK signaling pathway. Research has suggested that MAPK can regulate the epithelial-mesenchymal transition (EMT) process, thus impacting tumor metastasis (Huang et al., 2021b). Li et al. (2019) shown that METTL3 regulates the progression and migration of colorectal cancer by increasing the methylation level of SOX2 and enhancing the stability of methylated SOX2 through an m6A-IGF2BP2-dependent mechanism. It was also found that the tumor-suppressing METTL3 had low expression level in colorectal tumor, and it lost its inhibitory effect on p38/ERK in the MAPK signaling pathway and promoted colorectal cancer migration (Deng et al., 2019). In sum, METTL3 modulates the expression and function of various target molecules in a m6A-modified manner to affect the proliferation, apoptosis, metabolism, invasion and metastasis of colorectal cancer cells. Therefore, specific targeting of METTL3 has important application prospects in the treatment of colorectal cancer.

In contrast, another m6A writer protein, METTL14, expressed at a low level in colorectal cancer. The higher the expression of METTL14, the longer the overall survival of the patients, and overexpression of METTL14 can suppress the metastasis and progression of colorectal cancer (Chen et al., 2020b). METTL14 knockout was shown to reduce the level of m6A of its downstream target SOX4, resulting in reduced recognition of the modified SOX4 mRNA by YTHDF2, thus increasing SOX4 gene expression and promoting the SOX4-mediated EMT process and activity of the PI3K/AKT signaling pathway, leading to malignant progression of colorectal cancer (Chen et al., 2020a). This was also confirmed in another study in which the researcher found that the suppression of METTL14 can enhance the progression and metastasis of colorectal cancer (Wang et al., 2021). Tumor-associated macrophages (TAM) can inhibit the antitumor activity of T cells, but the mechanism is not clear. A recent study found that in colorectal cancer patients’ tumor tissue, the expression of METTL14 was negatively correlated with CD8^+^ T cell infiltration (Dong et al., 2021). Reduced expression of METTL14 in TAM subset C1q + cells can promote colon cancer cell growth and impede CD8^+^ T cell infiltration. It was further found that the reduction of Ebi3 expression level in C1q + cells can restore the anti-tumor killing ability of CD8^+^ T cells, while the loss of METTL14 expression can reduce the m6A modification level of Ebi3 mRNA in C1q + cells and promote the increase of its transcription level. Therefore, increasing the expression of METTL14 in TAM subset C1q + cells can promote CD8^+^ T cell infiltration and antitumor effects (Dong et al., 2021).

The third writer protein WTAP has been shown to have tumor-promoting action in many malignancies, including liver cancer, gastric cancer and osteosarcoma (Chen et al., 2019a; Li et al., 2020a; Chen et al., 2020c; Zhou et al., 2021b; Feng et al., 2021). In colorectal cancer, studies have pointed out WTAP is also oncogenic and can promote the progression of colorectal cancer through the WTAP/WT1/TBL1 axis in the canonical Wnt signaling pathway (Zhang et al., 2016a). Another group also suggested the higher expression of WTAP protein in colorectal cancer (Dong et al., 2022). In sum, both the dual role of METTL3 in colorectal tumors and the pro-oncogenic effect of METTL14 and WTAP in other tumors suggest that the m6A modification sites on different downstream target genes are different, which affect the activation status of various signaling pathways they are involved in, and thus play specific roles in tumors. Therefore, the next step in the investigation of m6A modification in the progression of colorectal tumor disease could be how to preserve the oncogenic role of the regulator by using inhibitors or developing new drugs to block its oncogenic signaling in specific signaling pathways.

However, unlike m6A methyltransferase, eraser and reader proteins have been less studied in colorectal cancer. It has been found that FTO is regulated by miR-1266 in the nucleus and initiates cell signaling molecules STAT3, cyclin D1 and MMPs to promote the development of colorectal cancer (Shen et al., 2018; Roslan et al., 2019; Ge et al., 2021). In addition, one group recently found that FTO is regulated by miR-96, which affects the methylation level of MYC and increase the expression of MYC, thus participating in the pro-proliferative and anti-apoptotic effects of miR-96 in colorectal cancer (Yue et al., 2020). While in the cytoplasm, FTO can dynamically regulate m6A modifications to contribute to the stemness of colorectal cancer cells and influence tumor drug resistance (Relier et al., 2021). Preliminary progress has also been made in the mechanism study of another demethylase, ALKBH5, in mediating colorectal cancer metastasis and immunotherapy resistance. The expression of ALKBH5 is downregulated in colon cancer and is associated with tumor metastasis, and it is pointed out that this molecule can be used as an independent predictor of patient prognosis (Yang et al., 2020). This study further confirmed the tumor suppressor effect of ALKBH5 both *in vitro* and *in vivo* (Yang et al., 2020). One study found that ALKBH5 can affect the tumor microenvironment of colorectal cancer, thus mediating the resistance of colorectal cancer patients to the anti-PD-1 therapy response (Li et al., 2020b). Meanwhile, ALKBH5 inhibitors can significantly improve immunotherapy efficacy, providing new possibilities for targeted therapy of colorectal cancer, melanoma, and other malignant tumors. Together, these demonstrated that ALKBH5 can not only be used as a breakthrough target for the clinical treatment of colorectal cancer in the future, but also has the potential to become a new biomarker for the patient population. Furthermore, there is still a gap in the study of the functional mechanisms of other members of the Alkb subfamily in colorectal tumors. The search for new molecules related to m6A modification and the study of their mechanisms is still a promising direction worth studying in the future.

Reader protein reads m6A modification sites of key molecules of colorectal tumor signaling pathways, affecting the expression level and RNA stability of these genes. In addition, the reader protein also forms complexes with noncoding RNAs related to colorectal tumors to improve its stability. Transcriptional and translational levels of YTHDF1 are significantly elevated in colorectal cancer patients. One study found YTHDF1 could enhance tumor cell stemness to promote tumorigenesis by inhibiting the canonical Wnt/β-catenin pathway (Bai et al., 2019). Another member of the YTHDF family, YTHDF3, affects colorectal cancer progression by regulating the negative feedback axis of the lncRNA GAS5-YAP-YTHDF3 in the Hippo pathway (Ni et al., 2019). Downregulation of the YTHDC2 gene can significantly inhibit the translation of tumor metastasis-related genes, such as hypoxia-inducible factor-1alpha (HIF-1α), and the high expression of this molecule is positively correlated with tumor stage and colon cancer metastasis (Tanabe et al., 2016).

**FIGURE 2:**
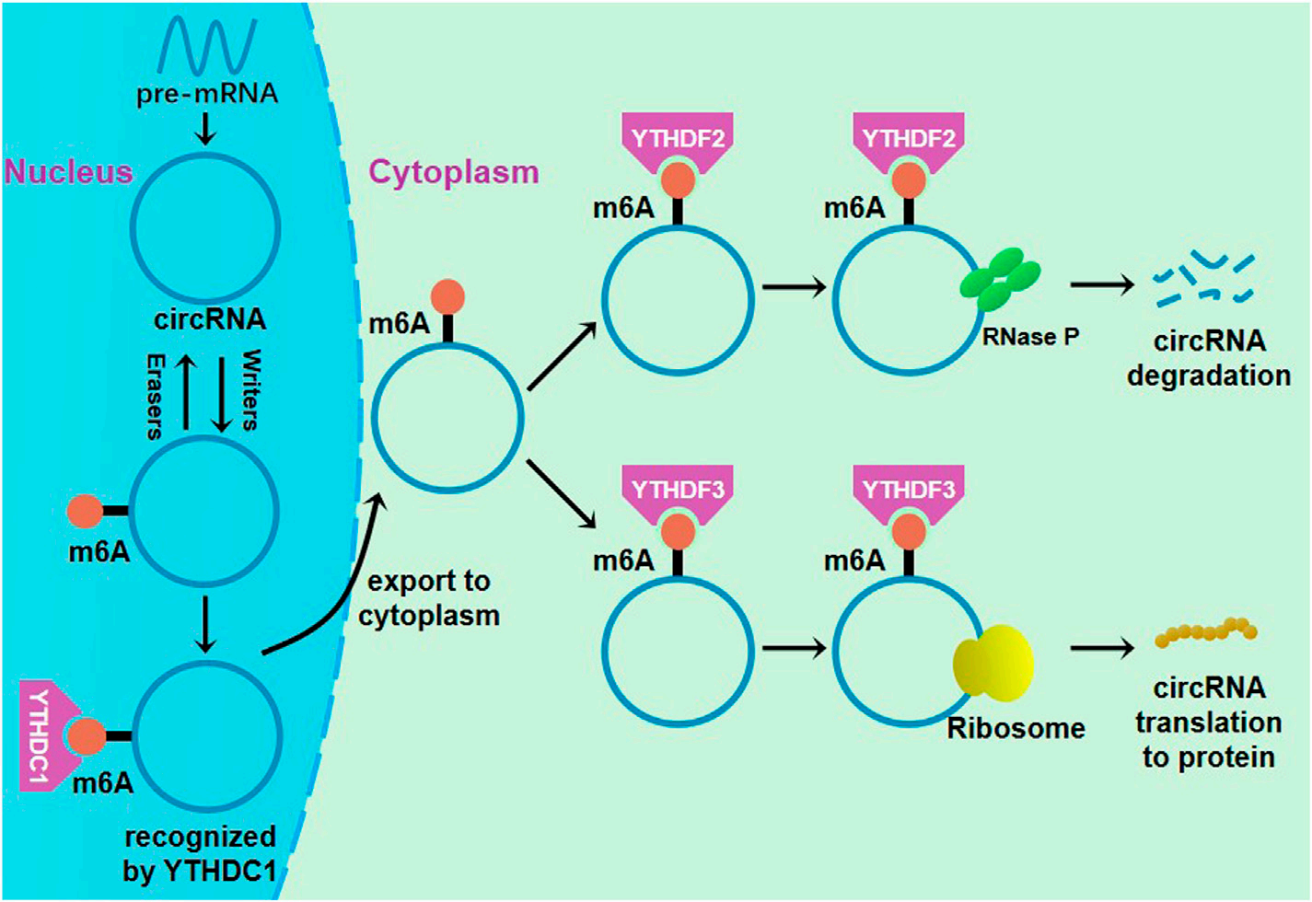
Regulation of m6A methylation in circRNA. YTHDC1 could promote circRNA nuclear export through binding to m6A methylation sites. The complex of eIF4G2, eIF4, eIF4B and YTHDF3 could initiate the translation of m6A-modified circRNA, and the circRNA could be cleaved by YTHDF2-HRSP12-RNase P/MRP complex.

circRNA is an RNA molecule with a novel structure. In recent years, modification of m6A has also been found in some circRNAs (Figure 2), with functions in the tumor occurrence and progression being reported (Zhao et al., 2019a; Li et al., 2021; Rao et al., 2021). In colorectal cancer, a study reported that circNSUN2 modified with m6A was frequently upregulated in tumor tissue and serum samples from patients with liver metastases of colorectal cancer (Chen et al., 2019b). The results show that after being recognized by YTHDC1, m6A-modified circNSUN2 will be exported to the cytoplasm, contributing to the formation of the ternary complex of circNSUN2-insulin-like growth factor 2 mRNA binding protein. circNSUN2 enhances the stability of HMGA2 mRNA, thereby promoting colorectal cancer invasion and liver metastasis (Chen et al., 2019b). circNSUN2 may become a potential therapeutic target for liver metastases of colorectal cancer.

### m5C Modification and Colorectal Cancer

**FIGURE 3:**
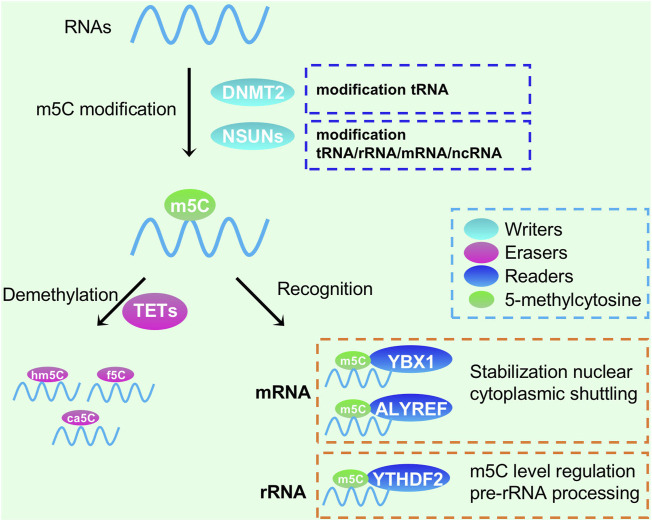
Functions of RNA m5C modification in RNA processing and metabolism. DNMT2 and NSUNs could methylate tRNA, rRNA, mRNA and ncRNA; YTHDF1, YBX1 and ALYREF could recognize m5C modification, while TETs could demethelate them.

m5C modifications are widely found in mRNA, rRNA, tRNA, and ncRNA, with the greatest abundance in tRNA and rRNA in eukaryotes (Figure 3). Enzymes responsible for RNA m5C modification include NSUN1 to NSUN7 of the NSUN family and DNA Methyltransferase-2 (DNMT2) (Bohnsack et al., 2019; Kuznetsova et al., 2019). NSUN1 and NSUN5 are involved in regulating 28S rRNA m5C modification, while NSUN3 and NSUN4 regulate mitochondrial tRNA and rRNA m5C modification, respectively. NSUN2 and DNMT2 regulate tRNA m5C modification in the cytoplasm, while NSUN7 targets eRNAs. The m5C regulator also functions in some digestive tumors. Studies have shown that the copy number of the NSUN2 gene is significantly increased in colorectal cancer, and the RNA methyltransferase NSUN2 is associated with the oncogene MYC. When the MYC protein is activated, the expression of NSUN2 is also upregulated (Alboushi et al., 2021). To date, no specific m5C methyltransferase inhibitor has been developed. However, studies have shown that azacytidine, as a novel antitumor drug, can reduce the proliferation ability of cancer cells by inhibiting DNMT2-mediated m5C modification, suggesting that reducing m5C modification of tRNA may be an effective cancer treatment strategy (Esteller and Pandolfi, 2017).

### m1A Modification and Colorectal Cancer

m1A modification means the attachment of a methyl group to the N1 position of RNA adenosine. The modification of m1A can significantly alter the structure of the RNA and the strength of protein-RNA interactions, thus affecting the protein translation process (Wiener and Schwartz, 2021). TRM10 and the TRM6-TRM61 complex mediate m1A modification of tRNA (Xiong et al., 2018; Zhang and Jia, 2018). The presence of m1A modification was also detected in 28S rRNA (Sloan et al., 2017). The YTH protein family can bind to m1A with low affinity, so it is considered as a potential m1A reader (Dai et al., 2018). ALKBH1 and ALKBH3, as demethylases, are involved in the regulation of m1A modification. Abnormal expression of m1A-related regulatory genes in digestive tumors is closely related to patient prognosis. Overexpression of ALKBH1 in colorectal cancer is one of the causative factors of a lower survival rate, as well as low expression of ALKBH3 (Zhao et al., 2019b).

### m7G Modification and Colorectal Cancer

The epigenetic modification m7G was originally found to exist within eukaryotic mRNA, tRNA, and rRNA. The most typical enzyme characterizing m7G methylation modification is METTL1. In tRNA, METTL1-WDR4 complex-mediated m7G modification maintains its structural integrity (Lin et al., 2018). The m7G in rRNA is mediated by the Williams-Beuren syndrome chromosome region 22 (WBSCR22), but its role is not fully understood. The m7G modification on rRNA may be involved in ribosome maturation, but has little effect on translation (Haag et al., 2015). The m7G modification within mRNA is enriched at the 5′UTR and is dynamically regulated with stress changes, and its role is to promote the translation process (Malbec et al., 2019). As an important regulator of m7G, METTL1 functions as a tumor suppressor in colorectal cancer (Liu et al., 2020). In addition, overexpression of METTL1 also enhance the chemosensitivity of colorectal cancer cells to cisplatin by regulating the miR-149-3p/S100A4/p53 axis (Liu et al., 2019). These results suggest that maintaining high levels of functional tRNA may be crucial for METTL1 executing function in cancer cells. Although the effect of METTL1 on tRNA may be cancer-promoting, there is no direct evidence that m7G modification plays a role in cancer cells. Another study demonstrated that WBSCR22 expression in colorectal cancer tissues is remarkably increased and upregulated WBSCR22 predicts a poor prognosis in patients (Yan et al., 2017). Knockdown of WBSCR22 can significantly improve the sensitivity of colorectal cancer cells to oxaliplatin, while overexpression of WBSCR22 increases cell resistance. Knockdown of WBSCR22 can increase oxaliplatin-induced intracellular ROS production and ROS-induced accumulation of 8-oxoguanine oxidative damage, which makes cancer cells more susceptible to oxaliplatin treatment (Yan et al., 2017).

### Pseudouridine (ψ) Modification and Colorectal Cancer

Pseudouridine is another abundant RNA modification in mRNA, tRNA, rRNA, snRNA, and lncRNA. In human cell lines, the proportion of pseudouridine (ψ) modifications in mRNA and lncRNA is about 30%–84% (Zhang et al., 2019). Pseudouridine is important in regulating the response to environmental stress. The presence of ψ can increase the rigidity of the RNA backbone, affecting its thermodynamic stability and spatial conformation, thus making the structure and function of the RNA more stable. ψ modification is involved in maintaining structural stability in tRNA and in the assembly of ribosomes in rRNA. One of the most studied diseases associated with defective pseudouridine modification is dyskeratosis congenita (DC) caused by inactivating mutations in pseudouridine synthase 1 (DKC1). Deficiency of DKC1 activity results in impaired telomerase activity and impaired mRNA translation, resulting in reduced cell replicative potential and premature senescence. Abnormal mutations in DKC1 are associated with various tumors such as colorectal cancer and hepatocellular carcinoma (McMahon, 2019). In colorectal cancer, reduced ψ modification on rRNA alters translation in cancer cells by affecting its interaction with the ribosomal P site (Babaian et al., 2020).

## Conclusions and Perspectives

RNA modification plays an important role as a key post-transcriptional regulator in gene expression, and researchers gradually realize that the functional network of its interaction involves many fields such as metabolism, epigenetics, chromatin remodeling, as well as the immune system. Although research on the epitranscriptome has made great progress, most studies have only focused on the biological functions of m6A modifications in mRNA, and the epitranscriptome includes more than 170 chemical modifications that modify coding and noncoding RNAs. Therefore, the association of hundreds of other RNA modifications with coding and noncoding RNAs remains to be explored. To explore the specific biological functions of various RNA modifications, it is first necessary to develop systematic methods and tools for rapid and quantitative detection of RNA modifications. Most existing sequencing methods are based on second generation sequencing technology, which cannot accurately identify chemical modifications on RNA. When researchers detect specific RNA modifications, they often need to detect RNA modifications using specific antibody-based immunoprecipitation techniques or indirect methods such as chemical labeling and the unique base modification properties of RNA pairings. Although these methods have good feasibility, its reproducibility is still low due to the reasons such as technology and incomplete algorithms. Therefore, future detection technologies and methods for various RNA modifications still need further research and exploration.

To date, treatments for colorectal cancer are still very limited. The rapid development of experimental technologies based on high-throughput sequencing and proteomics provides more possibilities for a deep study of the mechanism of RNA modification in colorectal cancer. In colorectal cancer, the complex of methyltransferase modified by m6A methylation, including METTL3, which plays a major catalytic role, has been thoroughly studied and is involved in the regulation of multiple classical signaling pathways in tumor development and apoptosis. However, there are still few studies on other writer molecules, and the roles of WTAP and METTL16, which can act independently, in colorectal tumors are still unclear. Similarly, eraser and reader proteins have been shown to contribute to the occurrence, development, metastasis and drug resistance of colorectal cancers by regulating classical signaling pathways, such as the Wnt pathway and the PI3K/AKT pathway, as well as changing the m6A modification level of noncoding RNAs, but there is still wide room for improvement in the transformation from basic to clinical. Although m6A methylation modification is abundant at the mRNA level, tumor-associated noncoding RNAs (miRNAs, lncRNAs and circRNAs) are also regulated by m6A methylation and act in the disease progression of colorectal cancer. circRNAs have recently been found to encode polypeptides. Whether m6A modification plays a key role and the specific mechanism has attracted the attention of researchers. Therefore, it is also essential to pay attention to tumor-associated noncoding RNAs. In addition, few eraser proteins have been found and only two more abundantly studied molecules, FTO and ALKBH5. ALKBH3 has been confirmed to have the function of demethylase, but there are still many areas that we have not studied in the Alk homolog family. It is still a research direction to explore whether more members of this family can function as demethylases. There are many types of reader proteins, and it is still unknown that reader proteins specifically read m6A modifications on different RNAs and play different downstream functions.

**FIGURE 4:**
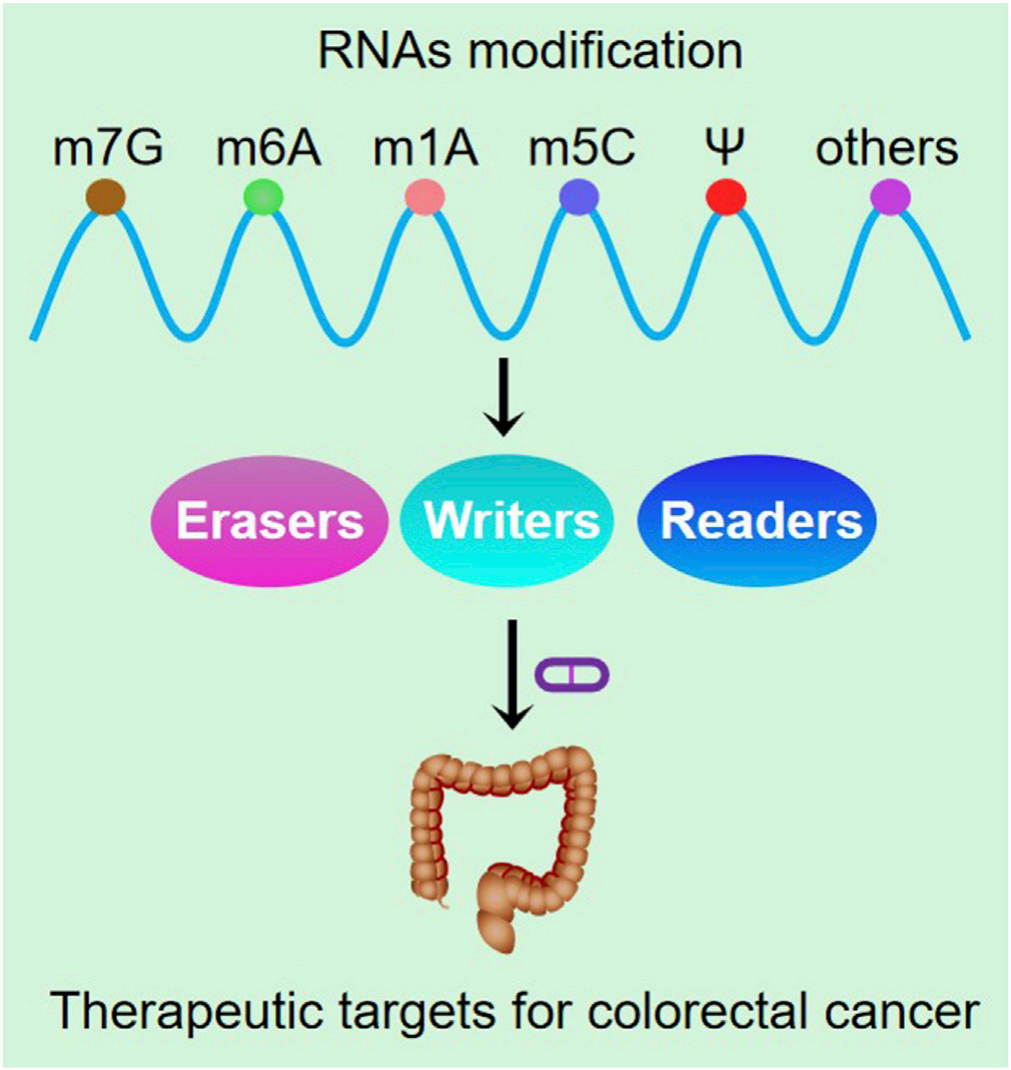
Schematic diagram of RNA methylation application in the clinic.

Various RNA modifications including m6A, m5C and ψ function in the regulation of stem cell function and cell survival under stress. The regulation of these RNA modifications may be of great value in reducing tumor cell chemotherapy resistance and tumor recurrence. The identification of precise epitranscriptomic biomarkers in a specific cell type or tumor microenvironment and the identification of aberrantly modified oncogenic or tumor suppressor effects are of great significance for finding precise molecular targets and developing highly selective and effective therapeutic approaches (Table 1). In the future, precision medicine based on epitranscriptomic signatures may be used for the diagnosis and treatment of colorectal cancer (Figure 4).

**TABLE 1:** The main factors of RNA modification.

Genes	Modification type	Target genes	Functions in cancer	References
Writer				
METTL3	m6A	MYC, EGFR, SP1, SP2, SOX2	Oncogene	Lin et al., (2016); Barbieri et al., (2017); Cui et al., (2017); Vu et al. (2017); Weng et al. (2018)
METTL14	m6A			
NSUNs	m5C	rRNA, NMR, HDGF	Oncogene	Saijo et al. (2001); Bantis et al. (2004); Li et al. (2018); Chen et al. (2019c)
METTL1	m7G	Pri-let7	Oncogene	Pandolfini et al. (2019)
PUS10	Pseudouridine	Unknown	Tumour suppressor	Jana et al. (2017)
DKC1	Pseudouridine	rRNA	Tumour suppressor	Ruggero et al. (2003); Montanaro et al. (2010)
		TERC	Oncogene	Penzo et al. (2015)
Eraser				
FTO	m6A	PDCD1, CXCR4, SOX10, ASB2, RARA	Oncogene	Iles et al. (2013); Li et al. (2017); Yang et al. (2019)
ALKBH5	m6A	FOXM1, NANOG	Oncogene	Zhang et al., (2016b); Zhang et al. (2017)
ALKBH3	m1A	tRNA, CSF1	Oncogene	Konishi et al. (2005); Chen et al. (2019d); Woo and Chambers, (2019)
Reader				
YTHDC2	m6A	HIF1A	Oncogene	Tanabe et al. (2016)
YTHDF1	m6A	FZD9, WNT6	Oncogene	Bai et al. (2019)
YTHDF2	m6A	TNFRSF1B	Oncogene	Paris et al. (2019)
IGF2BP1	m6A	SRF	Oncogene	Müller, (2019)
IGF2BP2	m6A	SOX2, MYC	Oncogene	Li et al. (2019); Wang et al. (2019)
